# Correction: Virulence and antimicrobial resistance profile of non-typhoidal *Salmonella enterica* serovars recovered from poultry processing environments at wet markets in Dhaka, Bangladesh

**DOI:** 10.1371/journal.pone.0272389

**Published:** 2022-07-27

**Authors:** PLOS ONE

The second, third, fourth, and fifth authors’ names are spelled incorrectly. The correct names are: Md Samun Sarker, Md. Shahidur Rahman Khan, Md. Tanvir Rahman, and Md. Abdul Kafi. The correct citation is: Siddiky NA, Sarker MS, Khan MSR, Rahman MT, Kafi MA, Samad MA (2022) Virulence and antimicrobial resistance profile of non-typhoidal *Salmonella enterica* serovars recovered from poultry processing environments at wet markets in Dhaka, Bangladesh. PLoS ONE 17(2): e0254465.

The publisher apologizes for the errors.
