# Supplementary material for: Virulence and antimicrobial resistance profile of non-typhoidal Salmonella enterica serovars recovered from poultry processing environments at wet markets in Dhaka, Bangladesh
Source: PLoS One. 2022 Feb 7;17(2):e0254465. doi: 10.1371/journal.pone.0254465 (PMC8820648; doi:10.1371/journal.pone.0254465)
Supplement: S1 Text — (DOCX) [file pone.0254465.s001.docx]

| **Table S1 A. Phenotypic and genotypic resistance patterns of all chopping board swab samples (chicken)** | | | | | |
| --- | --- | --- | --- | --- | --- |
| **Sl. No.** | **Lab ID** | **Chopping Board Swab (CBS)** | **Salmonella Enterica serovar** | **Phenotypic resistance** | **Genotypic resistance** |
|  |  |  |  |  |  |
| 1 | ARAC-CD-EN-1591 | Chopping board swab (chicken) | untyped | CIP-S-AMP-TE-NA-CN-AMC-C | blaTEM-sul1-TetA-StrA/B |
| 2 | ARAC-CD-EN-1594 | Chopping board swab (chicken) | S. Typhimurium | NA-SXT-AZM | sul1 |
| 3 | ARAC-CD-EN-1595 | Chopping board swab (chicken) | S. Typhimurium | CIP-S-AMP-TE-NA-CN-AMC-SXT | blaTEM-sul1-TetA-StrA/B |
| 4 | ARAC-CD-EN-1599 | Chopping board swab (chicken) | untyped | S-AMP-NA-AMC-SXT-C | blaTEM-sul1 |
| 5 | ARAC-CD-EN-1653 | Chopping board swab (chicken) | untyped | S-NA-AMC-SXT-C | sul1 |
| 6 | ARAC-CD-EN-1654 | Chopping board swab (chicken) | S. Typhimurium | CIP-S-AMP-TE-NA-CN-AMC | blaTEM-sul1-TetA |
| 7 | ARAC-CD-EN-1711 | Chopping board swab (chicken) | untyped | CIP-S-AMP-TE-NA-CN-SXT | blaTEM-sul1-TetA |
| 8 | ARAC-CD-EN-1714 | Chopping board swab (chicken) | S. Typhimurium | S-TE-AZM-SXT | sul1-TetA |
| 9 | ARAC-CD-EN-1771 | Chopping board swab (chicken) | untyped | AMP-NA-AZM | sul1 |
| 10 | ARAC-CD-EN-1773 | Chopping board swab (chicken) | S.Enteritidis | CIP-S-AMP-TE-NA-CN-AMC-SXT | blaTEM-sul1-TetA-StrA/B |
| 11 | ARAC-CD-EN-1776 | Chopping board swab (chicken) | S. Enteritidis | CIP-S-AMP-TE-NA--SXT | blaTEM-TetA-sul1 |
| 12 | ARAC-CD-EN-1893 | Chopping board swab (chicken) | S. Typhimurium | CIP-S-AMP-TE-NA-CN-AMC-SXT | blaTEM-sul1-TetA |
| 13 | ARAC-CD-EN-1951 | Chopping board swab (chicken) | S. Typhimurium | CIP-S-AMP-TE-NA-CN-AMC-C | blaTEM-sul1-TetA |
| 14 | ARAC-CD-EN-1952 | Chopping board swab (chicken) | S. Typhimurium | CIP-S-AMP-TE-NA-CN-AMC | blaTEM-sul1-TetA |
| 15 | ARAC-CD-EN-2011 | Chopping board swab (chicken) | S. Typhimurium | CIP-S-AMP-TE-NA-AMC-AZM | blaTEM-sul1-TetA-StrA/B |
| 16 | ARAC-CD-EN-2012 | Chopping board swab (chicken) | untyped | AZM | 0 |
| 17 | ARAC-CD-EN-2013 | Chopping board swab (chicken) | S. Enteritidis | NA | X |
| 18 | ARAC-CD-EN-2014 | Chopping board swab (chicken) | S. Typhimurium | CIP-S-AMP-TE-NA-CN-SXT-C | blaTEM-sul1-TetA |
| 19 | ARAC-CD-EN-2015 | Chopping board swab (chicken) | S. Typhimurium | CIP-S-AMP-TE-NA-CN-AMC | blaTEM-sul1-TetA |
| 20 | ARAC-CD-EN-2016 | Chopping board swab (chicken) | Unidentified | CIP-S-AMP-TE-NA-CN-SXT | blaTEM-sul1-TetA |
| 21 | ARAC-CD-EN-2071 | Chopping board swab (chicken) | S. Typhimurium | x | x |
| 22 | ARAC-CD-EN-2073 | Chopping board swab (chicken) | Unidentified | AMP-NA-SXT | blaTEM-sul1 |
| 23 | ARAC-CD-EN-2075 | Chopping board swab (chicken) | S. Typhimurium | AMP-NA | blaTEM |
| 24 | ARAC-CD-EN-2077 | Chopping board swab (chicken) | untyped | S-AMP-NA-SXT | blaTEM-sul1-StrA/B |
| 25 | ARAC-CD-EN-2078 | Chopping board swab (chicken) | untyped | CIP-AMP-AMC-SXT-C | blaTEM-sul1 |
| 26 | ARAC-CD-EN-2131 | Chopping board swab (chicken) | S. Enteritidis | CIP-S-AMP-TE-NA-SXT | blaTEM-sul1-TetA |
| 27 | ARAC-CD-EN-2132 | Chopping board swab (chicken) | S. Enteritidis | AZM | x |
| 28 | ARAC-CD-EN-2191 | Chopping board swab (chicken) | S. Typhimurium | CIP-S-AMP-TE-NA-CN-AZM | blaTEM-sul1-TetA-StrA/B |
| 29 | ARAC-CD-EN-2195 | Chopping board swab (chicken) | S. Typhimurium | S-AMP-TE-NA-CN-SXT-C | blaTEM-sul1-TetA |
| 30 | ARAC-CD-EN-2198 | Chopping board swab (chicken) | S. Typhimurium | CIP-S-AMP-TE-NA-CN-SXT-C | blaTEM-sul1-TetA |
| 31 | ARAC-CD-EN-2199 | Chopping board swab (chicken) | S. Typhimurium | CN | x |
| 32 | ARAC-CD-EN-2231 | Chopping board swab (chicken) | untyped | x | x |
| 33 | ARAC-CD-EN-2233 | Chopping board swab (chicken) | S. Typhimurium | CIP-S-AMP-TE-NA-CN-SXT-C | blaTEM-sul1-TetA-StrA/B |
| 34 | ARAC-CD-EN-2291 | Chopping board swab (chicken) | untyped | x | x |
| 35 | ARAC-CD-EN-2353 | Chopping board swab (chicken) | S. Typhimurium | x |  |
| 36 | ARAC-CD-EN-2354 | Chopping board swab (chicken) | S. Typhimurium | CIP-S-AMP-TE-NA-CN-SXT-C | blaTEM-sul1-TetA-StrA/B |
| 37 | ARAC-CD-EN-2357 | Chopping board swab (chicken) | S. Typhimurium | CIP-AMP-TE-NA-CN | blaTEM-sul1-TetA |
| 38 | ARAC-CD-EN-2411 | Chopping board swab (chicken) | S. Typhimurium | CIP-S-AMP-TE-NA-CN-AMC-SXT | blaTEM-sul1-sul3-StrA/B |
| 39 | ARAC-CD-EN-2412 | Chopping board swab (chicken) | S. Typhimurium | CIP-S-AMP-TE-NA-CN-AMC | blaTEM-sul1-TetA |
| 40 | ARAC-CD-EN-2471 | Chopping board swab (chicken) | S. Typhimurium | CIP-S-AMP-TE-NA-CN-SXT | blaTEM-sul1-TetA |
| 41 | ARAC-CD-EN-2472 | Chopping board swab (chicken) | untyped | x | x |
| 42 | ARAC-CD-EN-2532 | Chopping board swab (chicken) | S. Typhimurium | CIP-S-AMP-TE-NA-CN-AMC-AZM | blaTEM-sul1-TetA-StrA/B |
| 43 | ARAC-CD-EN-2594 | Chopping board swab (chicken) | S. Typhimurium | AMP | blaTEM |
| 44 | ARAC-CD-EN-2597 | Chopping board swab (chicken) | untyped | x | x |
| 45 | ARAC-CD-EN-2654 | Chopping board swab (chicken) | untyped | CIP-AMP-TE-NA-SXT | blaTEM-sul1-TetA |
| 46 | ARAC-CD-EN-2657 | Chopping board swab (chicken) | S. Typhimurium | CIP-S-TE-NA | blaTEM-TetA |
| 47 | ARAC-CD-EN-2660 | Chopping board swab (chicken) | untyped | CIP-S | 0 |
| 48 | ARAC-CD-EN-2712 | Chopping board swab (chicken) | S. Enteritidis | CIP-S | StrA/B |
| 49 | ARAC-CD-EN-2714 | Chopping board swab (chicken) | S. Typhimurium | CIP-S-AMP-TE-NA-CN-AMC-AZM-SXT | blaTEM-sul1-sul2-TetA |
| 50 | ARAC-CD-EN-2831 | Chopping board swab (chicken) | S. Typhimurium | CIP-S-AMP-TE-NA-CN | blaTEM-sul1-TetA |
| 51 | ARAC-CD-EN-2892 | Chopping board swab (chicken) | untyped | S | StrA/B |
| 52 | ARAC-CD-EN-2953 | Chopping board swab (chicken) | untyped | CIP-AMP-TE-NA-CN-AMC-SXT | blaTEM-sul2-TetA |
| 53 | ARAC-CD-EN-3015 | Chopping board swab (chicken) | untyped | CIP-TE-NA-AMC-SXT-CT-Ak | blaTEM-sul1-TetA-StrA/B |
| 54 | ARAC-CD-EN-3139 | Chopping board swab (chicken) | S. Typhimurium | CIP-S-AMP-TE-NA-CN-AMC-SXT | blaTEM-sul1-TetA |
| 55 | ARAC-CD-EN-3191 | Chopping board swab (chicken) | untyped | CIP-S-AMP-TE-CN-C | blaTEM-sul1-TetA |
| 56 | ARAC-CD-EN-3192 | Chopping board swab (chicken) | S. Typhimurium | CIP-S-AMP-TE-CN-AMC-CRO | blaTEM-sul1-TetA-StrA/B |

**Table S1 B. Phenotypic and genotypic resistance patterns of all carcass dressing water samples**

| **Sl. No.** | **Lab ID** | **Carcass dressing water (CDW)** | **Salmonella Enterica serovars** | **Phenotypic Resistance** | **Genotypic Resistance** |
| --- | --- | --- | --- | --- | --- |
|  |  |  |  |  |  |
| 1 | ARAC-CD-EN-1554 | Carcass dressing water (chicken) | untyped | CIP-AMP-TE-NA-MEM | blaTEM-TetA |
| 2 | ARAC-CD-EN-1611 | Carcass dressing water (chicken) | S. Enteritidis | CIP-NA-CN-SXT-AK | blaTEM |
| 3 | ARAC-CD-EN-1620 | Carcass dressing water (chicken) | untyped | CIP-S-AMP-TE-NA-CN-AMC | blaTEM-sul1-TetA-StrA/B |
| 4 | ARAC-CD-EN-1671 | Carcass dressing water (chicken) | S. Enteritidis | CIP-AMP-TE-SXT-C | blaTEM-TetA |
| 5 | ARAC-CD-EN-1733 | Carcass dressing water (chicken) | untyped | S-AMP-NA-SXT-C | blaTEM-sul1-TetA |
| 6 | ARAC-CD-EN-1800 | Carcass dressing water (chicken) | untyped | CIP-AMP-TE-NA-C | blaTEM-sul1-TetA |
| 7 | ARAC-CD-EN-1853 | Carcass dressing water (chicken) | S. Typhimurium | CIP-TE-NA-CN-AMC | blaTEM-sul1-TetA |
| 8 | ARAC-CD-EN-1854 | Carcass dressing water (chicken) | untyped | S-AMC | x |
| 9 | ARAC-CD-EN-1855 | Carcass dressing water (chicken) | S. Enteritidis | CIP-S-AMP-TE-NA-CN-AMC | blaTEM-sul1-TetA-StrA/B |
| 10 | ARAC-CD-EN-1859 | Carcass dressing water (chicken) | S. Typhimurium | CIP-AMP-CN-AMC-AZM | blaTEM-sul1-TetA-StrA/B |
| 11 | ARAC-CD-EN-1860 | Carcass dressing water (chicken) | S. Typhimurium | CIP-S-AMP-TE-NA-CN | blaTEM-sul1-TetA |
| 12 | ARAC-CD-EN-1913 | Carcass dressing water (chicken) | S. Typhimurium | CIP-S-AMP-TE-NA-CN | blaTEM-sul1-TetA |
| 13 | ARAC-CD-EN-1971 | Carcass dressing water (chicken) | untyped | CIP-S-AMP-TE-NA-CN-AMC | blaTEM-sul1-TetA |
| 14 | ARAC-CD-EN-1973 | Carcass dressing water (chicken) | S. Typhimurium | CIP-S-AMP-TE-NA-CN-AMC-SXT-CT-MEM | blaTEM-sul1-TetA-StrA/B |
| 15 | ARAC-CD-EN-1974 | Carcass dressing water (chicken) | S. Typhimurium | CIP-S-AMP-TE-NA-CN-AMC | blaTEM-sul1-TetA |
| 16 | ARAC-CD-EN-2091 | Carcass dressing water (chicken) | untyped | CIP-S-AMP-TE-NA-CN-SXT-C | blaTEM-sul1-TetA-StrA/B |
| 17 | ARAC-CD-EN-2092 | Carcass dressing water (chicken) | untyped | CIP-NA | TetA-StrA/B |
| 18 | ARAC-CD-EN-2093 | Carcass dressing water (chicken) | S. Typhimurium | S-TE | blaTEM-sul1-TetA |
| 19 | ARAC-CD-EN-2094 | Carcass dressing water (chicken) | Unidentified | CIP | 0 |
| 20 | ARAC-CD-EN-2095 | Carcass dressing water (chicken) | S. Typhimurium | CIP-AMP-TE-C | blaTEM-sul1-TetA |
| 21 | ARAC-CD-EN-2096 | Carcass dressing water (chicken) | S. Typhimurium | AMP-TE-NA-C | blaTEM |
| 22 | ARAC-CD-EN-2097 | Carcass dressing water (chicken) | untyped | S-AMP-NA-SXT-C | sul1-StrA/B |
| 23 | ARAC-CD-EN-2151 | Carcass dressing water (chicken) | S. Typhimurium | x | blaTEM-TetA |
| 24 | ARAC-CD-EN-2154 | Carcass dressing water (chicken) | Unidentified | S-AMP-TE-NA-SXT | sul1-StrA/B |
| 25 | ARAC-CD-EN-2158 | Carcass dressing water (chicken) | S. Typhimurium | CIP-S-AMP-TE-NA-CN-AZM | blaTEM-TetA-StrA/B |
| 26 | ARAC-CD-EN-2211 | Carcass dressing water (chicken) | untyped | x | 0 |
| 27 | ARAC-CD-EN-2213 | Carcass dressing water (chicken) | untyped | S-AMP-TE-NA-SXT-C | sul1-StrA/B |
| 28 | ARAC-CD-EN-2215 | Carcass dressing water (chicken) | untyped | AMP-NA-SXT-C | sul1 |
| 29 | ARAC-CD-EN-2216 | Carcass dressing water (chicken) | untyped | x | 0 |
| 30 | ARAC-CD-EN-2217 | Carcass dressing water (chicken) | S. Enteritidis | AMP-NA | blaTEM |
| 31 | ARAC-CD-EN-2218 | Carcass dressing water (chicken) | Unidentified | S | StrA/B |
| 32 | ARAC-CD-EN-2219 | Carcass dressing water (chicken) | S. Typhimurium | CIP-S-AMP-TE-NA-C | blaTEM-sul1-TetA |
| 33 | ARAC-CD-EN-2220 | Carcass dressing water (chicken) | untyped | x | 0 |
| 34 | ARAC-CD-EN-2251 | Carcass dressing water (chicken) | untyped | CIP-S-TE-NA | 0 |
| 35 | ARAC-CD-EN-2258 | Carcass dressing water (chicken) | S. Typhimurium | CIP-S-AMP-TE-NA-CN-AMC-SXT-C | blaTEM-sul1-TetA-StrA/B |
| 36 | ARAC-CD-EN-2311 | Carcass dressing water (chicken) | Unidentified | AMC | TetA |
| 37 | ARAC-CD-EN-2371 | Carcass dressing water (chicken) | Unidentified | CIP-C | 0 |
| 38 | ARAC-CD-EN-2372 | Carcass dressing water (chicken) | S. Typhimurium | CIP-AMP-TE-NA-CN | blaTEM-sul1-TetA |
| 39 | ARAC-CD-EN-2373 | Carcass dressing water (chicken) | S. Typhimurium | CIP-S-AMP-TE-NA-CN | blaTEM-sul1-TetA |
| 40 | ARAC-CD-EN-2374 | Carcass dressing water (chicken) | untyped | CIP-S-AMP-TE-NA-CN-SXT | blaTEM-sul1-TetA-StrA/B |
| 41 | ARAC-CD-EN-2432 | Carcass dressing water (chicken) | S. Typhimurium | CIP-S-AMP-TE-NA-CN-AMC | blaTEM-sul1-StrA/B |
| 42 | ARAC-CD-EN-2434 | Carcass dressing water (chicken) | S. Typhimurium | CIP-S-AMP-TE-NA-CN-AMC | blaTEM-sul1-TetA-StrA/B |
| 43 | ARAC-CD-EN-2491 | Carcass dressing water (chicken) | S. Typhimurium | CIP-S-AMP-TE-NA-CN-AMC | blaTEM-sul1-TetA |
| 44 | ARAC-CD-EN-2620 | Carcass dressing water (chicken) | S. Typhimurium | CIP-S-AMP-TE-NA-CN-AMC | blaTEM-sul1-TetA-StrA/B |
| 45 | ARAC-CD-EN-2674 | Carcass dressing water (chicken) | untyped | CIP-S-CT | 0 |
| 46 | ARAC-CD-EN-2675 | Carcass dressing water (chicken) | untyped | CIP-S-AMP-TE-NA-CN-AMC-SXT-CT-AK | blaTEM-sul1-TetA-StrA/B |
| 47 | ARAC-CD-EN-2678 | Carcass dressing water (chicken) | untyped | S | 0 |
| 48 | ARAC-CD-EN-2731 | Carcass dressing water (chicken) | untyped | AMP-TE-NA-AZM | 0 |
| 49 | ARAC-CD-EN-2733 | Carcass dressing water (chicken) | untyped | CIP-S | x |
| 50 | ARAC-CD-EN-2740 | Carcass dressing water (chicken) | S. Typhimurium | CIP-S-TE-AMC-CT | blaTEM-sul1-TetA-StrA/B |
| 51 | ARAC-CD-EN-2912 | Carcass dressing water (chicken) | untyped | CIP-S-AZM | TetA |
| 52 | ARAC-CD-EN-2920 | Carcass dressing water (chicken) | S. Typhimurium | CIP-S-AMP-TE-NA-CN-AMC-SXT | blaTEM-sul1-TetA |
| 53 | ARAC-CD-EN-2974 | Carcass dressing water (chicken) | untyped | CIP | x |
| 54 | ARAC-CD-EN-2980 | Carcass dressing water (chicken) | S. Enteritidis | CIP-S-AMC-SXT | blaTEM-sul1-StrA/B |
| 55 | ARAC-CD-EN-3033 | Carcass dressing water (chicken) | S. Typhimurium | CIP-S-AMP-TE-NA-CN | blaTEM-sul1-TetA |
| 56 | ARAC-CD-EN-3155 | Carcass dressing water (chicken) | S. Typhimurium | CIP-TE | blaTEM-sul1-TetA |
| 57 | ARAC-CD-EN-3213 | Carcass dressing water (chicken) | untyped | TE-NA-SXT | sul1-TetA |
| 58 | ARAC-CD-EN-3214 | Carcass dressing water (chicken) | untyped | CIP-S-TE-SXT-CT | sul1-TetA-StrA/B |

**Table S1 C. Phenotypic and genotypic resistance patterns of all Knife swab samples**

| Sl. No. | Lab ID | Knife Swab (KS) | Salmonella Enterica serovar | Phenotypic resistance | Genotypic resistance |
| --- | --- | --- | --- | --- | --- |
|  |  |  |  |  |  |
| 1 | ARAC-CD-EN-1541 | Knife swab | S. Typhimurium | CIP-S-AMP-TE-NA-CN-AZM | blaTEM-TetA-StrA/B |
| 2 | ARAC-CD-EN-1603 | Knife swab | untyped | AMP-TE-NA-MEM | blaTEM-TetA |
| 3 | ARAC-CD-EN-1606 | Knife swab | S. Enteritidis | S-TE-NA-AMC-SXT-C-AK | TetA-sul1 |
| 4 | ARAC-CD-EN-1669 | Knife swab | S. Typhimurium | CIP-S-AMP-TE-NA-CN-AMC-AZM-SXT | blaTEM-sul1-TetA |
| 5 | ARAC-CD-EN-1781 | Knife swab | S. Typhimurium | CIP-S-AMP-TE-NA-CN-AMC-C-SXT | blaTEM-sul1-sul3-TetA-StrA/B |
| 6 | ARAC-CD-EN-1783 | Knife swab | S. Typhimurium | CIP-S-AMP-TE-NA-CN-AMC-C-SXT | blaTEM-sul1-TetA-StrA/B |
| 7 | ARAC-CD-EN-1784 | Knife swab | S. Typhimurium | CIP-S-AMP-TE-NA-CN-AMC-C-SXT | blaTEM-sul1-sul3-TetA |
| 8 | ARAC-CD-EN-1841 | Knife swab | untyped | CIP-S-AMP-TE-NA-CN | TetA-StrA/B |
| 9 | ARAC-CD-EN-1902 | Knife swab | S. Typhimurium | MEM | x |
| 10 | ARAC-CD-EN-1903 | Knife swab | S. Typhimurium | CIP-S-AMP-TE-NA-CN-AMC-C-SXT | blaTEM-sul1-TetA |
| 11 | ARAC-CD-EN-1963 | Knife swab | S. Typhimurium | CIP-S-AMP-TE-NA-CN-AMC | blaTEM-sul1-TetA-StrA/B |
| 12 | ARAC-CD-EN-1964 | Knife swab | S. Typhimurium | CIP-S-AMP-TE-NA-CN-AMC-SXT-C | blaTEM-sul1-TetA-StrA/B |
| 13 | ARAC-CD-EN-1968 | Knife swab | S. Typhimurium | AMP-TE-NA-CN-AMC-SXT-C-AZM- | blaTEM-sul1-sul3-TetA |
| 14 | ARAC-CD-EN-2022 | Knife swab | S. Typhimurium | CIP-S-AMP-TE-NA-SXT | blaTEM-sul1-sul3-TetA |
| 15 | ARAC-CD-EN-2081 | Knife swab | S. Typhimurium | C | x |
| 16 | ARAC-CD-EN-2082 | Knife swab | S. Enteritidis | AMP-NA | blaTEM |
| 17 | ARAC-CD-EN-2083 | Knife swab | S. Typhimurium | CIP-AMP-TE-NA-CN-SXT | blaTEM-sul1-TetA |
| 18 | ARAC-CD-EN-2084 | Knife swab | S. Typhimurium | CIP-S-AMP-TE-NA-CN | blaTEM-sul1-TetA |
| 19 | ARAC-CD-EN-2085 | Knife swab | untyped | S-AMP-NA-SXT-C | blaTEM-sul1 |
| 20 | ARAC-CD-EN-2086 | Knife swab | S. Typhimurium | CIP-S-AMP-TE-NA-CN-SXT | blaTEM-sul1-TetA-StrA/B |
| 21 | ARAC-CD-EN-2087 | Knife swab | untyped | AMP-NA | blaTEM-sul1 |
| 22 | ARAC-CD-EN-2088 | Knife swab | S. Typhimurium | SXT | sul1 |
| 23 | ARAC-CD-EN-2141 | Knife swab | S. Typhimurium | CIP-S-AMP-TE-NA-CN-SXT | blaTEM-sul1-TetA |
| 24 | ARAC-CD-EN-2142 | Knife swab | Unidentified | x | 0 |
| 25 | ARAC-CD-EN-2143 | Knife swab | Unidentified | S-AMP-TE-SXT | blaTEM-sul3-TetA-StrA/B |
| 26 | ARAC-CD-EN-2146 | Knife swab | Unidentified | CIP-SXT | sul1 |
| 27 | ARAC-CD-EN-2201 | Knife swab | Unidentified | S-AMP-TE-NA-CN-SXT-C-AK | blaTEM-TetA |
| 28 | ARAC-CD-EN-2204 | Knife swab | S. Typhimurium | CIP-S-AMP-TE-NA-CN-SXT-C | blaTEM-sul1-sul3-TetA |
| 29 | ARAC-CD-EN-2207 | Knife swab | untyped | CN | x |
| 30 | ARAC-CD-EN-2243 | Knife swab | untyped | x | x |
| 31 | ARAC-CD-EN-2249 | Knife swab | untyped | x | x |
| 32 | ARAC-CD-EN-2301 | Knife swab | untyped | AMC | x |
| 33 | ARAC-CD-EN-2361 | Knife swab | untyped | CIP-S-AMP-TE-NA-CN | blaTEM-TetA-StrA/B |
| 34 | ARAC-CD-EN-2364 | Knife swab | S. Typhimurium | CIP-S-AMP-TE-NA-CN-SXT | blaTEM-TetA-StrA/B |
| 35 | ARAC-CD-EN-2423 | Knife swab | S. Typhimurium | CIP-AMP-TE-NA | blaTEM-TetA |
| 36 | ARAC-CD-EN-2481 | Knife swab | S. Typhimurium | CIP-S-AMP-TE-NA-CN-AMC | blaTEM-TetA-StrA/B |
| 37 | ARAC-CD-EN-2487 | Knife swab | S. Typhimurium | CIP-S-AMP-TE-NA-CN-AMC-SXT | blaTEM-sul1-TetA |
| 38 | ARAC-CD-EN-2542 | Knife swab | S. Typhimurium | CIP-S-AMP-TE-NA-CN-AMC | blaTEM-sul1-TetA |
| 39 | ARAC-CD-EN-2543 | Knife swab | S. Typhimurium | CIP |  |
| 40 | ARAC-CD-EN-2544 | Knife swab | S. Typhimurium | CIP-S-AMP-TE-NA-CN-SXT | blaTEM-sul1-TetA |
| 41 | ARAC-CD-EN-2664 | Knife swab | untyped | S-MEM | 0 |
| 42 | ARAC-CD-EN-2723 | Knife swab | untyped | CIP | 0 |
| 43 | ARAC-CD-EN-2901 | Knife swab | untyped | CIP-S | 0 |
| 44 | ARAC-CD-EN-2903 | Knife swab | untyped | S-AZM-CAZ | 0 |
| 45 | ARAC-CD-EN-2905 | Knife swab | untyped | CIP-S-CT | sul1 |
| 46 | ARAC-CD-EN-2967 | Knife swab | untyped | CIP-S-AMP-TE-NA-AMC-SXT-CT-CAZ-CRO-ATM | blaTEM-sul3-TetA-StrA/B |
| 47 | ARAC-CD-EN-3024 | Knife swab | untyped | CIP-S-AMP-AMC | blaTEM-StrA/B |
| 48 | ARAC-CD-EN-3028 | Knife swab | untyped | CIP-CT-CRO-ATM-SXT | sul1 |
| 49 | ARAC-CD-EN-3142 | Knife swab | untyped | CIP-S | 0 |
| 50 | ARAC-CD-EN-3146 | Knife swab | untyped | CIP-S-AMP-AMC-CT-CAZ-SXT-ATM | blaTEM-sul3-TetA-StrA/B |
| 51 | ARAC-CD-EN-3201 | Knife swab | untyped | CIP-S-AMP-TE-NA-CN-AMC-SXT-C-AK | blaTEM-sul3-TetA-StrA/B |
