# Supplementary material for: Virulence and antimicrobial resistance profile of non-typhoidal Salmonella enterica serovars recovered from poultry processing environments at wet markets in Dhaka, Bangladesh
Source: PLoS One. 2022 Feb 7;17(2):e0254465. doi: 10.1371/journal.pone.0254465 (PMC8820648; doi:10.1371/journal.pone.0254465)
Supplement: S2 Text — (DOCX) [file pone.0254465.s002.docx]

**Table S2 A**. AMR patterns and MAR index of *Salmonella* *enterica* serovars isolated from CDW

| Isolate No. | Serovars | Phenotypic Pattern | Genotypic Pattern | MAR Index |
| --- | --- | --- | --- | --- |
| ARAC-CD-EN-1554 | Untyped | CIP-AMP-TE-NA-MEM | blaTEM-TetA | 0.31 |
| ARAC-CD-EN-1611 | S. Enteritidis | CIP-NA-CN-SXT-AK | blaTEM | 0.31 |
| ARAC-CD-EN-1620 | Untyped | CIP-S-AMP-TE-NA-CN-AMC | blaTEM-sul1-TetA-StrA/B | 0.43 |
| ARAC-CD-EN-1671 | S. Enteritidis | CIP-AMP-TE-SXT-C | blaTEM-TetA | 0.31 |
| ARAC-CD-EN-1733 | Untyped | S-AMP-NA-SXT-C | blaTEM-sul1-TetA | 0.31 |
| ARAC-CD-EN-1800 | Untyped | CIP-AMP-TE-NA-C | blaTEM-sul1-TetA | 0.31 |
| ARAC-CD-EN-1853 | S. Typhimurium | CIP-TE-NA-CN-AMC | blaTEM-sul1-TetA | 0.31 |
| ARAC-CD-EN-1854 | Untyped | S-AMC | - | 0.12 |
| ARAC-CD-EN-1855 | S. Enteritidis | CIP-S-AMP-TE-NA-CN-AMC | blaTEM-sul1-TetA-StrA/B | 0.43 |
| ARAC-CD-EN-1859 | S. Typhimurium | CIP-AMP-CN-AMC-AZM | blaTEM-sul1-TetA-StrA/B | 0.31 |
| ARAC-CD-EN-1860 | S. Typhimurium | CIP-S-AMP-TE-NA-CN | blaTEM-sul1-TetA | 0.37 |
| ARAC-CD-EN-1913 | S. Typhimurium | CIP-S-AMP-TE-NA-CN | blaTEM-sul1-TetA | 0.37 |
| ARAC-CD-EN-1971 | Untyped | CIP-S-AMP-TE-NA-CN-AMC | blaTEM-sul1-TetA | 0.43 |
| ARAC-CD-EN-1973 | S. Typhimurium | CIP-S-AMP-TE-NA-CN-AMC-SXT-CT-MEM | blaTEM-sul1-TetA-StrA/B | 0.62 |
| ARAC-CD-EN-1974 | S. Typhimurium | CIP-S-AMP-TE-NA-CN-AMC | blaTEM-sul1-TetA | 0.43 |
| ARAC-CD-EN-2091 | Untyped | CIP-S-AMP-TE-NA-CN-SXT-C | blaTEM-sul1-TetA-StrA/B | 0.5 |
| ARAC-CD-EN-2092 | Untyped | CIP-NA | TetA-StrA/B | 0.12 |
| ARAC-CD-EN-2093 | S. Typhimurium | S-TE | blaTEM-sul1-TetA | 0.12 |
| ARAC-CD-EN-2094 | Untyped | CIP | - | 0.06 |
| ARAC-CD-EN-2095 | S. Typhimurium | CIP-AMP-TE-C | blaTEM-sul1-TetA | 0.25 |
| ARAC-CD-EN-2096 | S. Typhimurium | AMP-TE-NA-C | blaTEM | 0.25 |
| ARAC-CD-EN-2097 | Untyped | S-AMP-NA-SXT-C | sul1-StrA/B | 0.31 |
| ARAC-CD-EN-2151 | S. Typhimurium | - | blaTEM-TetA | - |
| ARAC-CD-EN-2154 | Untyped | S-AMP-TE-NA-SXT | sul1-StrA/B | 0.31 |
| ARAC-CD-EN-2158 | S. Typhimurium | CIP-S-AMP-TE-NA-CN-AZM | blaTEM-TetA-StrA/B | 0.43 |
| ARAC-CD-EN-2211 | Untyped | - | - | - |
| ARAC-CD-EN-2213 | Untyped | S-AMP-TE-NA-SXT-C | sul1-StrA/B | 0.37 |
| ARAC-CD-EN-2215 | Untyped | AMP-NA-SXT-C | sul1 | 0.25 |
| ARAC-CD-EN-2216 | Untyped | - | - | - |
| ARAC-CD-EN-2217 | S. Enteritidis | AMP-NA | blaTEM | 0.12 |
| ARAC-CD-EN-2218 | Untyped | S | StrA/B | 0.06 |
| ARAC-CD-EN-2219 | S. Typhimurium | CIP-S-AMP-TE-NA-C | blaTEM-sul1-TetA | 0.37 |
| ARAC-CD-EN-2220 | Untyped | - | - | - |
| ARAC-CD-EN-2251 | Untyped | CIP-S-TE-NA | - | 0.25 |
| ARAC-CD-EN-2258 | S. Typhimurium | CIP-S-AMP-TE-NA-CN-AMC-SXT-C | blaTEM-sul1-TetA-StrA/B | 0.56 |
| ARAC-CD-EN-2311 | Untyped | AMC | TetA | 0.06 |
| ARAC-CD-EN-2371 | Untyped | CIP-C | - | 0.12 |
| ARAC-CD-EN-2372 | S. Typhimurium | CIP-AMP-TE-NA-CN | blaTEM-sul1-TetA | 0.31 |
| ARAC-CD-EN-2373 | S. Typhimurium | CIP-S-AMP-TE-NA-CN | blaTEM-sul1-TetA | 0.37 |
| ARAC-CD-EN-2374 | Untyped | CIP-S-AMP-TE-NA-CN-SXT | blaTEM-sul1-TetA-StrA/B | 0.43 |
| ARAC-CD-EN-2432 | S. Typhimurium | CIP-S-AMP-TE-NA-CN-AMC | blaTEM-sul1-StrA/B | 0.43 |
| ARAC-CD-EN-2434 | S. Typhimurium | CIP-S-AMP-TE-NA-CN-AMC | blaTEM-sul1-TetA-StrA/B | 0.43 |
| ARAC-CD-EN-2491 | S. Typhimurium | CIP-S-AMP-TE-NA-CN-AMC | blaTEM-sul1-TetA | 0.43 |
| ARAC-CD-EN-2620 | S. Typhimurium | CIP-S-AMP-TE-NA-CN-AMC | blaTEM-sul1-TetA-StrA/B | 0.43 |
| ARAC-CD-EN-2674 | Untyped | CIP-S-CT | - | 0.18 |
| ARAC-CD-EN-2675 | Untyped | CIP-S-AMP-TE-NA-CN-AMC-SXT-CT-AK | blaTEM-sul1-TetA-StrA/B | 0.62 |
| ARAC-CD-EN-2678 | Untyped | S | - | 0.06 |
| ARAC-CD-EN-2731 | Untyped | AMP-TE-NA-AZM | - | 0.25 |
| ARAC-CD-EN-2733 | Untyped | CIP-S | - | 0.12 |
| ARAC-CD-EN-2740 | S. Typhimurium | CIP-S-TE-AMC-CT | blaTEM-sul1-TetA-StrA/B | 0.31 |
| ARAC-CD-EN-2912 | Untyped | CIP-S-AZM | TetA | 0.18 |
| ARAC-CD-EN-2920 | S. Typhimurium | CIP-S-AMP-TE-NA-CN-AMC-SXT | blaTEM-sul1-TetA | 0.5 |
| ARAC-CD-EN-2974 | Untyped | CIP | - | 0.06 |
| ARAC-CD-EN-2980 | S. Enteritidis | CIP-S-AMC-SXT | blaTEM-sul1-StrA/B | 0.25 |
| ARAC-CD-EN-3033 | S. Typhimurium | CIP-S-AMP-TE-NA-CN | blaTEM-sul1-TetA | 0.31 |
| ARAC-CD-EN-3155 | S. Typhimurium | CIP-TE | blaTEM-sul1-TetA | 0.12 |
| ARAC-CD-EN-3213 | Untyped | TE-NA-SXT | sul1-TetA | 0.18 |
| ARAC-CD-EN-3214 | Untyped | CIP-S-TE-SXT-CT | sul1-TetA-StrA/B | 0.31 |

**Table S2 B**. AMR patterns and MAR index of *Salmonella enterica* serovars isolated from CBS

| Isolate No. | Serovars | Phenotypic pattern | Genotypic Pattern | MAR Index |
| --- | --- | --- | --- | --- |
| ARAC-CD-EN-1591 | Untyped | CIP-S-AMP-TE-NA-CN-AMC-C | blaTEM-sul1-TetA-StrA/B | 0.5 |
| ARAC-CD-EN-1594 | S. Typhimurium | NA-SXT-AZM | sul1 | 0.18 |
| ARAC-CD-EN-1595 | S. Typhimurium | CIP-S-AMP-TE-NA-CN-AMC-SXT | blaTEM-sul1-TetA-StrA/B | 0.5 |
| ARAC-CD-EN-1599 | Untyped | S-AMP-NA-AMC-SXT-C | blaTEM-sul1 | 0.37 |
| ARAC-CD-EN-1653 | Untyped | S-NA-AMC-SXT-C | sul1 | 0.31 |
| ARAC-CD-EN-1654 | S. Typhimurium | CIP-S-AMP-TE-NA-CN-AMC | blaTEM-sul1-TetA | 0.43 |
| ARAC-CD-EN-1711 | Untyped | CIP-S-AMP-TE-NA-CN-SXT | blaTEM-sul1-TetA | 0.43 |
| ARAC-CD-EN-1714 | S. Typhimurium | S-TE-AZM-SXT | sul1-TetA | 0.25 |
| ARAC-CD-EN-1771 | Untyped | AMP-NA-AZM | sul1 | 0.18 |
| ARAC-CD-EN-1773 | S. Enteritidis | CIP-S-AMP-TE-NA-CN-AMC-SXT | blaTEM-sul1-TetA-StrA/B | 0.5 |
| ARAC-CD-EN-1776 | S. Enteritidis | CIP-S-AMP-TE-NA--SXT | blaTEM-TetA-sul1 | 0.37 |
| ARAC-CD-EN-1893 | S. Typhimurium | CIP-S-AMP-TE-NA-CN-AMC-SXT | blaTEM-sul1-TetA | 0.5 |
| ARAC-CD-EN-1951 | S. Typhimurium | CIP-S-AMP-TE-NA-CN-AMC-C | blaTEM-sul1-TetA | 0.43 |
| ARAC-CD-EN-1952 | S. Typhimurium | CIP-S-AMP-TE-NA-CN-AMC | blaTEM-sul1-TetA | 0.43 |
| ARAC-CD-EN-2011 | S. Typhimurium | CIP-S-AMP-TE-NA-AMC-AZM | blaTEM-sul1-TetA-StrA/B | 0.43 |
| ARAC-CD-EN-2012 | Unidentified | AZM | - | 0.06 |
| ARAC-CD-EN-2013 | S. Enteritidis | NA | - | 0.06 |
| ARAC-CD-EN-2014 | S. Typhimurium | CIP-S-AMP-TE-NA-CN-SXT-C | blaTEM-sul1-TetA | 0.5 |
| ARAC-CD-EN-2015 | S. Typhimurium | CIP-S-AMP-TE-NA-CN-AMC | blaTEM-sul1-TetA | 0.43 |
| ARAC-CD-EN-2016 | Untyped | CIP-S-AMP-TE-NA-CN-SXT | blaTEM-sul1-TetA | 0.43 |
| ARAC-CD-EN-2071 | S. Typhimurium | - | - | - |
| ARAC-CD-EN-2073 | Untyped | AMP-NA-SXT | blaTEM-sul1 | 0.18 |
| ARAC-CD-EN-2075 | S. Typhimurium | AMP-NA | blaTEM | 0.12 |
| ARAC-CD-EN-2077 | Untyped | S-AMP-NA-SXT | blaTEM-sul1-StrA/B | 0.25 |
| ARAC-CD-EN-2078 | Untyped | CIP-AMP-AMC-SXT-C | blaTEM-sul1 | 0.31 |
| ARAC-CD-EN-2131 | S. Enteritidis | CIP-S-AMP-TE-NA-SXT | blaTEM-sul1-TetA | 0.37 |
| ARAC-CD-EN-2132 | S. Enteritidis | AZM | - | 0.06 |
| ARAC-CD-EN-2191 | S. Typhimurium | CIP-S-AMP-TE-NA-CN-AZM | blaTEM-sul1-TetA-StrA/B | 0.43 |
| ARAC-CD-EN-2195 | S. Typhimurium | S-AMP-TE-NA-CN-SXT-C | blaTEM-sul1-TetA | 0.43 |
| ARAC-CD-EN-2198 | S. Typhimurium | CIP-S-AMP-TE-NA-CN-SXT-C | blaTEM-sul1-TetA | 0.5 |
| ARAC-CD-EN-2199 | S. Typhimurium | CN | - | 0.06 |
| ARAC-CD-EN-2231 | Unidentified | - | - | - |
| ARAC-CD-EN-2233 | S. Typhimurium | CIP-S-AMP-TE-NA-CN-SXT-C | blaTEM-sul1-TetA-StrA/B | 0.5 |
| ARAC-CD-EN-2291 | Untyped | - | - | - |
| ARAC-CD-EN-2353 | S. Typhimurium | - | - | - |
| ARAC-CD-EN-2354 | S. Typhimurium | CIP-S-AMP-TE-NA-CN-SXT-C | blaTEM-sul1-TetA-StrA/B | 0.5 |
| ARAC-CD-EN-2357 | S. Typhimurium | CIP-AMP-TE-NA-CN | blaTEM-sul1-TetA | 0.31 |
| ARAC-CD-EN-2411 | S. Typhimurium | CIP-S-AMP-TE-NA-CN-AMC-SXT | blaTEM-sul1-sul3-StrA/B | 0.5 |
| ARAC-CD-EN-2412 | S. Typhimurium | CIP-S-AMP-TE-NA-CN-AMC | blaTEM-sul1-TetA | 0.43 |
| ARAC-CD-EN-2471 | S. Typhimurium | CIP-S-AMP-TE-NA-CN-SXT | blaTEM-sul1-TetA | 0.43 |
| ARAC-CD-EN-2472 | Unidentified | - | - | - |
| ARAC-CD-EN-2532 | S. Typhimurium | CIP-S-AMP-TE-NA-CN-AMC-AZM | blaTEM-sul1-TetA-StrA/B | 0.5 |
| ARAC-CD-EN-2594 | S. Typhimurium | AMP | blaTEM | 0.06 |
| ARAC-CD-EN-2597 | Untyped | - | - | - |
| ARAC-CD-EN-2654 | Untyped | CIP-AMP-TE-NA-SXT | blaTEM-sul1-TetA | 0.31 |
| ARAC-CD-EN-2657 | S. Typhimurium | CIP-S-TE-NA | blaTEM-TetA | 0.25 |
| ARAC-CD-EN-2660 | Untyped | CIP-S | - | 0.12 |
| ARAC-CD-EN-2712 | S. Enteritidis | CIP-S | StrA/B | 0.12 |
| ARAC-CD-EN-2714 | S. Typhimurium | CIP-S-AMP-TE-NA-CN-AMC-AZM-SXT | blaTEM-sul1-sul2-TetA | 0.56 |
| ARAC-CD-EN-2831 | S. Typhimurium | CIP-S-AMP-TE-NA-CN | blaTEM-sul1-TetA | 0.37 |
| ARAC-CD-EN-2892 | Untyped | S | StrA/B | 0.06 |
| ARAC-CD-EN-2953 | Untyped | CIP-AMP-TE-NA-CN-AMC-SXT | blaTEM-sul2-TetA | 0.43 |
| ARAC-CD-EN-3015 | Untyped | CIP-TE-NA-AMC-SXT-CT-AK | blaTEM-sul1-TetA-StrA/B | 0.43 |
| ARAC-CD-EN-3139 | S. Typhimurium | CIP-S-AMP-TE-NA-CN-AMC-SXT | blaTEM-sul1-TetA | 0.5 |
| ARAC-CD-EN-3191 | Untyped | CIP-S-AMP-TE-CN-C | blaTEM-sul1-TetA | 0.37 |
| ARAC-CD-EN-3192 | S. Typhimurium | CIP-S-AMP-TE-CN-AMC-CRO | blaTEM-sul1-TetA-StrA/B | 0.43 |

**Table S2 C.** AMR patterns and MAR index of *Salmonella enterica* serovars isolated from KS

| Isolate No. | Serovars | Phenotypic pattern | Genotypic Pattern | MAR Index |
| --- | --- | --- | --- | --- |
| ARAC-CD-EN-1541 | S. Typhimurium | CIP-S-AMP-TE-NA-CN-AZM | blaTEM-TetA-StrA/B | 0.43 |
| ARAC-CD-EN-1603 | S. Enteritidis | AMP-TE-NA-MEM | blaTEM-TetA | 0.25 |
| ARAC-CD-EN-1606 | S. Enteritidis | S-TE-NA-AMC-SXT-C-AK | TetA-sul1 | 0.43 |
| ARAC-CD-EN-1669 | S. Typhimurium | CIP-S-AMP-TE-NA-CN-AMC-AZM-SXT | blaTEM-sul1-TetA | 0.56 |
| ARAC-CD-EN-1781 | S. Typhimurium | CIP-S-AMP-TE-NA-CN-AMC-C-SXT | blaTEM-sul1-sul3-TetA-StrA/B | 0.56 |
| ARAC-CD-EN-1783 | S. Typhimurium | CIP-S-AMP-TE-NA-CN-AMC-C-SXT | blaTEM-sul1-TetA-StrA/B | 0.56 |
| ARAC-CD-EN-1784 | S. Typhimurium | CIP-S-AMP-TE-NA-CN-AMC-C-SXT | blaTEM-sul1-sul3-TetA | 0.56 |
| ARAC-CD-EN-1841 | Untyped | CIP-S-AMP-TE-NA-CN | TetA-StrA/B | 0.37 |
| ARAC-CD-EN-1902 | S. Typhimurium | MEM | - | 0.06 |
| ARAC-CD-EN-1903 | S. Typhimurium | CIP-S-AMP-TE-NA-CN-AMC-C-SXT | blaTEM-sul1-TetA | 0.56 |
| ARAC-CD-EN-1963 | S. Typhimurium | CIP-S-AMP-TE-NA-CN-AMC | blaTEM-sul1-TetA-StrA/B | 0.43 |
| ARAC-CD-EN-1964 | S. Typhimurium | CIP-S-AMP-TE-NA-CN-AMC-SXT-C | blaTEM-sul1-TetA-StrA/B | 0.56 |
| ARAC-CD-EN-1968 | S. Typhimurium | AMP-TE-NA-CN-AMC-SXT-C-AZM- | blaTEM-sul1-sul3-TetA | 0.5 |
| ARAC-CD-EN-2022 | S. Typhimurium | CIP-S-AMP-TE-NA-SXT | blaTEM-sul1-sul3-TetA | 0.37 |
| ARAC-CD-EN-2081 | S. Typhimurium | CN | - | 0.06 |
| ARAC-CD-EN-2082 | S. Enteritidis | AMP-NA | blaTEM | 0.12 |
| ARAC-CD-EN-2083 | S. Typhimurium | CIP-AMP-TE-NA-CN-SXT | blaTEM-sul1-TetA | 0.37 |
| ARAC-CD-EN-2084 | S. Typhimurium | CIP-S-AMP-TE-NA-CN | blaTEM-sul1-TetA | 0.37 |
| ARAC-CD-EN-2085 | Unidentified | S-AMP-NA-SXT-C | blaTEM-sul1 | 0.31 |
| ARAC-CD-EN-2086 | S. Typhimurium | CIP-S-AMP-TE-NA-CN-SXT | blaTEM-sul1-TetA-StrA/B | 0.43 |
| ARAC-CD-EN-2087 | Unidentified | AMP-NA | blaTEM-sul1 | 0.12 |
| ARAC-CD-EN-2088 | S. Typhimurium | SXT | sul1 | 0.06 |
| ARAC-CD-EN-2141 | S. Typhimurium | CIP-S-AMP-TE-NA-CN-SXT | blaTEM-sul1-TetA | 0.43 |
| ARAC-CD-EN-2142 | Untyped | - | - | - |
| ARAC-CD-EN-2143 | Untyped | S-AMP-TE-SXT | blaTEM-sul3-TetA-StrA/B | 0.25 |
| ARAC-CD-EN-2146 | Untyped | CIP-SXT | sul1 | 0.12 |
| ARAC-CD-EN-2201 | Untyped | S-AMP-TE-NA-CN-SXT-C-AK | blaTEM-TetA | 0.5 |
| ARAC-CD-EN-2204 | S. Typhimurium | CIP-S-AMP-TE-NA-CN-SXT-C | blaTEM-sul1-sul3-TetA | 0.5 |
| ARAC-CD-EN-2207 | Untyped | CN | - | 0.06 |
| ARAC-CD-EN-2243 | Untyped | - | - | - |
| ARAC-CD-EN-2249 | Untyped | - | - | - |
| ARAC-CD-EN-2301 | Untyped | AMC | - | 0.06 |
| ARAC-CD-EN-2361 | Untyped | CIP-S-AMP-TE-NA-CN | blaTEM-TetA-StrA/B | 0.37 |
| ARAC-CD-EN-2364 | S. Typhimurium | CIP-S-AMP-TE-NA-CN-SXT | blaTEM-TetA-StrA/B | 0.43 |
| ARAC-CD-EN-2423 | S. Typhimurium | CIP-AMP-TE-NA | blaTEM-TetA | 0.25 |
| ARAC-CD-EN-2481 | S. Typhimurium | CIP-S-AMP-TE-NA-CN-AMC | blaTEM-TetA-StrA/B | 0.43 |
| ARAC-CD-EN-2487 | S. Typhimurium | CIP-S-AMP-TE-NA-CN-AMC-SXT | blaTEM-sul1-TetA | 0.5 |
| ARAC-CD-EN-2542 | S. Typhimurium | CIP-S-AMP-TE-NA-CN-AMC | blaTEM-sul1-TetA | 0.43 |
| ARAC-CD-EN-2543 | S. Typhimurium | CIP | - | 0.06 |
| ARAC-CD-EN-2544 | S. Typhimurium | CIP-S-AMP-TE-NA-CN-SXT | blaTEM-sul1-TetA | 0.43 |
| ARAC-CD-EN-2664 | Untyped | S-MEM | - | 0.12 |
| ARAC-CD-EN-2723 | Untyped | CIP | - | 0.06 |
| ARAC-CD-EN-2901 | Untyped | CIP-S | - | 0.12 |
| ARAC-CD-EN-2903 | Untyped | S-AZM-CAZ | - | 0.18 |
| ARAC-CD-EN-2905 | Untyped | CIP-S-CT | sul1 | 0.18 |
| ARAC-CD-EN-2967 | Untyped | CIP-S-AMP-TE-NA-AMC-SXT-CT-CAZ-CRO-ATM | blaTEM-sul3-TetA-StrA/B | 0.68 |
| ARAC-CD-EN-3024 | Untyped | CIP-S-AMP-AMC | blaTEM-StrA/B | 0.25 |
| ARAC-CD-EN-3028 | Untyped | CIP-CT-CRO-ATM-SXT | sul1 | 0.31 |
| ARAC-CD-EN-3142 | Untyped | CIP-S | - | 0.12 |
| ARAC-CD-EN-3146 | Untyped | CIP-S-AMP-AMC-CT-CAZ-SXT-ATM | blaTEM-sul3-TetA-StrA/B | 0.5 |
| ARAC-CD-EN-3201 | Untyped | CIP-S-AMP-TE-NA-CN-AMC-SXT-C-AK | blaTEM-sul3-TetA-StrA/B | 0.56 |
