# Supplementary material for: Virulence and antimicrobial resistance profile of non-typhoidal Salmonella enterica serovars recovered from poultry processing environments at wet markets in Dhaka, Bangladesh
Source: PLoS One. 2022 Feb 7;17(2):e0254465. doi: 10.1371/journal.pone.0254465 (PMC8820648; doi:10.1371/journal.pone.0254465)
Supplement: S3 Text — (DOCX) [file pone.0254465.s003.docx]

**Table S3 A.** Statistical associations of antimicrobial resistance pattern among the carcass dressing water, chopping board swab and knife swab

| Antimicrobials | CIP | S | AMP | TE | NA | CN | SXT | AMC | C | AZM | AK | MEM | ATM | CRO | CT | CAZ | P- value |
| --- | --- | --- | --- | --- | --- | --- | --- | --- | --- | --- | --- | --- | --- | --- | --- | --- | --- |
| CDW | 40 | 33 | 34 | 35 | 36 | 23 | 16 | 18 | 12 | 4 | 2 | 2 | 0 | 0 | 4 | 0 | P<0.0001 |
| KS | 33 | 33 | 33 | 28 | 30 | 24 | 24 | 14 | 11 | 4 | 2 | 3 | 3 | 2 | 4 | 3 |  |
| CBS | 35 | 36 | 38 | 34 | 30 | 26 | 26 | 20 | 11 | 8 | 1 | 0 | 0 | 0 | 0 | 0 |  |

**Table S3 B.** Statistical associations of resistance patterns among the antimicrobials in carcass dressing water (CDW)

| Antimicrobial | CIP | S | AMP | TE | NA | CN | SXT | AMC | C | AZM | AK | MEM | ATM | CRO | CT | CAZ | p-value |
| --- | --- | --- | --- | --- | --- | --- | --- | --- | --- | --- | --- | --- | --- | --- | --- | --- | --- |
|  |  |  |  |  |  |  |  |  |  |  |  |  |  |  |  |  |  |
| Resistance | 40 | 33 | 34 | 35 | 36 | 23 | 16 | 18 | 12 | 4 | 2 | 2 | 0 | 0 | 4 | 0 | p<0.0001 |
| Susceptible | 18 | 25 | 24 | 23 | 22 | 35 | 42 | 40 | 46 | 54 | 56 | 56 | 58 | 58 | 54 | 58 |  |

**Table S3 C.** Statistical associations of resistance patterns among the *Salmonella enterica* serovars in carcass dressing water (CDW)

| Antimicrobials | CIP | S | AMP | TE | NA | CN | SXT | AMC | C | AZM | AK | MEM | ATM | CRO | CT | CAZ | P-value |
| --- | --- | --- | --- | --- | --- | --- | --- | --- | --- | --- | --- | --- | --- | --- | --- | --- | --- |
|  |  |  |  |  |  |  |  |  |  |  |  |  |  |  |  |  |  |
| *S.* Typhimurium | 20 | 16 | 18 | 20 | 17 | 16 | 3 | 11 | 4 | 2 | 0 | 1 | 0 | 0 | 2 | 0 | P<0.0001 |
| *S*. Enteritidis | 4 | 2 | 3 | 2 | 3 | 2 | 3 | 2 | 1 | 0 | 1 | 0 | 0 | 0 | 0 | 0 |  |
| Untyped *Salmonella* | 16 | 15 | 13 | 13 | 16 | 5 | 10 | 5 | 7 | 2 | 1 | 1 | 0 | 0 | 2 | 0 |  |
| Susceptible | 18 | 25 | 24 | 23 | 22 | 35 | 42 | 40 | 46 | 54 | 56 | 56 | 58 | 58 | 54 | 58 |  |

**Table S3 D.** Statistical associations of resistance patterns among the antimicrobials in chopping board swab (CBS)

| Antimicrobials | CIP | S | AMP | TE | NA | CN | SXT | AMC | C | AZM | AK | MEM | ATM | CRO | CT | CAZ | p-value |
| --- | --- | --- | --- | --- | --- | --- | --- | --- | --- | --- | --- | --- | --- | --- | --- | --- | --- |
|  |  |  |  |  |  |  |  |  |  |  |  |  |  |  |  |  |  |
| Resistance | 35 | 36 | 38 | 34 | 30 | 26 | 26 | 20 | 11 | 8 | 1 | 0 | 0 | 0 | 0 | 0 | P<0.0001 |
| Susceptible | 21 | 20 | 18 | 22 | 26 | 30 | 30 | 36 | 45 | 48 | 55 | 56 | 56 | 56 | 56 | 56 |  |

**Table S3 E.** Statistical associations of resistance patterns among the *Salmonella enterica* serovars in chopping board swab (CBS)

| Antimicrobials | CIP | S | AMP | TE | NA | CN | SXT | AMC | C | AZM | AK | MEM | ATM | CRO | CT | CAZ | p- value |
| --- | --- | --- | --- | --- | --- | --- | --- | --- | --- | --- | --- | --- | --- | --- | --- | --- | --- |
|  |  |  |  |  |  |  |  |  |  |  |  |  |  |  |  |  |  |
| S. Typhimurium | 22 | 23 | 24 | 24 | 23 | 22 | 13 | 13 | 6 | 6 | 0 | 0 | 0 | 0 | 0 | 0 | P<0.0001 |
| S. Enteritidis | 4 | 4 | 3 | 3 | 4 | 1 | 3 | 1 | 0 | 1 | 0 | 0 | 0 | 0 | 0 | 0 |  |
| Untyped | 9 | 9 | 11 | 7 | 3 | 7 | 10 | 6 | 5 | 1 | 1 | 0 | 0 | 0 | 0 | 0 |  |
| Susceptible | 21 | 20 | 18 | 22 | 26 | 30 | 30 | 36 | 45 | 48 | 55 | 56 | 56 | 56 | 56 | 56 |  |

**Table S3 F.** Statistical associations of resistance patterns among the antimicrobials in knife swab (KS)

| Antimicrobials | CIP | S | AMP | TE | NA | CN | SXT | AMC | C | AZM | AK | MEM | ATM | CRO | CT | CAZ | P- value |
| --- | --- | --- | --- | --- | --- | --- | --- | --- | --- | --- | --- | --- | --- | --- | --- | --- | --- |
|  |  |  |  |  |  |  |  |  |  |  |  |  |  |  |  |  |  |
| Resistance | 33 | 33 | 33 | 28 | 30 | 24 | 24 | 14 | 11 | 4 | 2 | 3 | 3 | 2 | 4 | 3 | P<0.0001 |
| Susceptible | 18 | 18 | 18 | 23 | 21 | 27 | 27 | 37 | 40 | 47 | 49 | 48 | 48 | 49 | 47 | 48 |  |

**Table S3 G.** Statistical associations of resistance patterns among the *Salmonella enterica* serovars in knife swab KS)

| Antimicrobials | CIP | S | AMP | TE | NA | CN | SXT | AMC | C | AZM | AK | MEM | ATM | CRO | CT | CAZ | p- value |
| --- | --- | --- | --- | --- | --- | --- | --- | --- | --- | --- | --- | --- | --- | --- | --- | --- | --- |
|  |  |  |  |  |  |  |  |  |  |  |  |  |  |  |  |  |  |
| *S*. Typhimurium | 22 | 18 | 21 | 21 | 21 | 19 | 16 | 11 | 8 | 3 | 0 | 1 | 0 | 0 | 1 | 0 | P<0.0001 |
| *S*. Enteritidis | 0 | 1 | 2 | 2 | 3 | 0 | 1 | 1 | 1 | 0 | 1 | 1 | 0 | 0 | 0 | 0 |  |
| Untyped *Salmonella* | 11 | 14 | 10 | 5 | 6 | 5 | 7 | 2 | 2 | 1 | 1 | 1 | 3 | 2 | 3 | 3 |  |
| Susceptible | 18 | 18 | 18 | 23 | 21 | 27 | 27 | 37 | 40 | 47 | 49 | 48 | 48 | 49 | 47 | 48 |  |

**Table S3 H.** Statistical associations of genotypic resistance patterns among the *Salmonella enterica* serovars in CDW

| Genotypic resistance | *bla*TEM | *Tet*A | *Sul*1 | *Str*A/B | p- value |
| --- | --- | --- | --- | --- | --- |
| *S*. Typhimurium | 23 | 21 | 20 | 8 | P<0.0001 |
| *S*. Enteritidis | 5 | 2 | 2 | 2 |  |
| Untyped *Salmonella* | 8 | 12 | 13 | 11 |  |

**Table S3 I.** Statistical associations of genotypic resistance patterns among the *Salmonella enterica* serovars in CBS

| Genotypic resistance | *bla*TEM | *Tet*A | *Sul*1 | *Sul*2 | *Sul*3 | StrA/B | P- value |
| --- | --- | --- | --- | --- | --- | --- | --- |
| *S*. Typhimurium | 24 | 23 | 24 | 1 | 1 | 8 | p<0.0001 |
| *S*. Entertidis | 3 | 3 | 3 | 0 | 0 | 2 |  |
| Untyped *Salmonella* | 12 | 7 | 12 | 1 | 1 | 4 |  |

**Table S3 J.** Statistical associations of genotypic resistance patterns among the *Salmonella enterica* serovars in KS

| Genotypic resistance | *bla*TEM | *Tet*A | *Sul*1 | *Sul*3 | *Str*A/B | p- value |
| --- | --- | --- | --- | --- | --- | --- |
| *S.* Typhimurium | 22 | 21 | 19 | 5 | 8 | p<0.0001 |
| *S*. Entertidis | 2 | 2 | 1 | 0 | 0 |  |
| Untyped *Salmonella* | 8 | 7 | 5 | 4 | 8 |  |

**Table S3 K.** Statistical associations among the virulence genes of CDW, CBS and KS

| Prevalence of virulence genes (%) | | | | | | | | | | P- value |
| --- | --- | --- | --- | --- | --- | --- | --- | --- | --- | --- |
| **Samples** | **Serovars** | ***Inv*A** | ***Agf*A** | ***Ipf*A** | ***Hil*A** | ***Siv*H** | ***SopE*** | ***SefA*** | ***Spv*C** |  |
| CDW | *S.* Typhimurium | 23 | 23 | 23 | 30 | 30 | 30 | 0 | 0 | p<0.0001 |
|  | *S. Enteritidis* | 5 | 5 | 5 | 30 | 30 | 30 | 30 | 30 |  |
|  | untyped *Salmonella* | 30 | 30 | 30 | 30 | 30 | 30 | 30 | 30 |  |
| CBS | *S.* Typhimurium | 30 | 30 | 30 | 30 | 30 | 30 | 0 | 0 | p<0.0001 |
|  | *S. Enteritidis* | 6 | 6 | 6 | 6 | 6 | 6 | 6 | 6 |  |
|  | untyped *Salmonella* | 20 | 20 | 20 | 20 | 20 | 20 | 20 | 20 |  |
| KS | *S.* Typhimurium | 25 | 25 | 25 | 25 | 25 | 25 | 0 | 0 | p<0.0001 |
|  | *S. Enteritidis* | 3 | 3 | 3 | 3 | 3 | 3 | 3 | 3 |  |
|  | untyped *Salmonella* | 23 | 23 | 23 | 23 | 23 | 23 | 23 | 23 |  |
