# Supplementary material for: Virulence and antimicrobial resistance profile of non-typhoidal Salmonella enterica serovars recovered from poultry processing environments at wet markets in Dhaka, Bangladesh
Source: PLoS One. 2022 Feb 7;17(2):e0254465. doi: 10.1371/journal.pone.0254465 (PMC8820648; doi:10.1371/journal.pone.0254465)
Supplement: Attachment — Submitted filename: Responses to reviewers.docx [file pone.0254465.s004.docx]

Kumar Venkitanarayanan, DVM, Ph.D.
Academic Editor
PLOS ONE
Ref. No.: PONE-D-21-19124

Title: Virulence and antimicrobial resistance profile of non-typhoidal *Salmonella enterica* serovars recovered from poultry processing environments at wet markets in Dhaka, Bangladesh

Dear Editor,

We would like to submit the revised manuscripts herewith after incorporating the responses of the two reviewers for your kind consideration and further necessary action. Please have a look at the answers of the reviewers' comments.

Best regards,

Mohammed A. Samad

Corresponding author

**Responses to editor and reviewer’s comments**

| **Editor Comments** | | |
| --- | --- | --- |
| **Editor comments** | **Author responses** | **Remarks** |
| Please ensure that your manuscript meets PLOS ONE's style requirements, including those for file naming | The style of PLOS ONE has been followed as per given guidelines of the journal | Reflected in the text. |
| PLOS requires an ORCID ID for the corresponding author | The ORCID ID for corresponding author is included  (0000-0001-7329-7325) | ORCID ID Included |
| Copyright for Figure 1(Map) | The map (figure 1) is created for this study by using longitude and latitude data. Indeed, the copyright is ours | The map is created for this study |
| **Reviewer #1:** This manuscript is suitable for publication following suggested modifications | | |
| **Reviewers comments** | **Author responses** | **Remarks** |
| Manuscript requires a lot of editing and revision as there were a lot of issues related grammatical structure and phrasing in the scientific context. | The professional linguistic editing has been made in the whole manuscript where it is appropriate | Necessary correction made and highlighted in the whole manuscript |
| Improve the description in the statistical analysis section | The description of the statistical section has been improved as suggested by the reviewer | Line number: 226-229 |
| MAR index calculation in this study | The detail method of MAR index calculation is included in the material & method section | Line number: 194-199 |
| **Reviewer #2:** This manuscript is suitable for publication following suggested modifications | | |
| **Reviewers comments** | **Author responses** | **Remarks** |
| The manuscript was poorly written with many sentences either copied and pasted or rearranged from another article published by the same author | The English editing has been done in the whole manuscript and rewrite/ rephrase the sentences which is highlighted by the yellow color in the text. | Necessary correction made and highlighted in the whole manuscript |
| There were numerous grammatical errors throughout the manuscript | The grammatical error has corrected throughout the manuscript | Necessary correction made and highlighted in the whole manuscript |
| Several statements were mentioned repeatedly. For example: Poultry as a source of Salmonella infections has been repeatedly mentioned. | The repeated statement has been reshuffled | Line number:  60-61 |
| Lines 92 -103: Plagiarism noted from another article from the same author | The sentences has been modified or reshuffled | Line number:  92-106 |
| Please explain the number of samples collected per site. Were samples collected on a single visit for each of the site? | The ten samples of each three types (CDW, CBS and KS) were collected from each site in a single visit.  The sampling frequency and time has been added in the “study design and sample collection” section as suggested by the reviewer. | Line number:  134-135 |
| Figure 1: Title should explain the inset and the pointers marked in the figure | The title of figure-1 is modified in line with reviewer comments. The point is marked with blue color. | Line number: 141 |
| Plagiarism- Lines 149-150, 156 – 160 | The sentences has been reshuffled/modified and or replaced | Line number: 149-157 and 159-165 |
| Table 1: Please include the target gene names | The target gene name is included in the table 1 | Reflected in Table 1 (212-213 page) |
| Please explain MAR | MAR is explained in the manuscript | Line number: 194-199 |
| It would be better to represent Figure 2A-C as a table with the percentage of resistance | Figure 2A-C has modified into table (Table 3, 4 and 5); Similarly, figure 3 modified to table (Table 6, 7 and 8) | Table 3, 4 and 5; Table 6, 7 and 8 |
| sul3 gene was detected in both KS and CBS. Please correct line 279 | It is corrected in the text | Line number:  304-306 |
| Please explain “There was a strong correlation exist among the virulence genes in CDW, CBS, and KS (p < 0.005)” (line 293). It would be better to give figure 4 as a table as it is difficult to understand the results and, to identify the correlation between samples. | The analysis showed significantly higher associations among the virulence genes in CDW, CBS and KS (p < 0.005). The detail statistical analysis is given in Supplementary file 2 (Table 11).  Figure 4 has converted to Table 9 as suggested by the reviewer | Line Number:  326-328  Table 9 |
| Please rewrite the discussion section to contain only relevant data pertaining to the results | The discussion section has been modified as suggested by the reviewer | Reflected in the discussion section  Line: 332-528 |
| 416- 419-Plagiarism noted | The statements are modified and reshuffled | Line number: 438-444 |
| Please add reference for the statement Line 475 | The reference (Chmielewski and Frank, 2003) is included in the text as suggested | Line number: 643-645 |
| Line 344 – “Furthermore, China and some European countries detected S. Enteritidis and S. Typhimurium as the most prevalent serotypes” Did not explain from where they detected the serotypes | The sources are catering point and meat of pork, chicken and duck. The information is included in the text. | Line number:  379-381 |
